# Nursing Progress and Its Developments: VI. The Probationer's First Two Years

**Published:** 1918-01-26

**Authors:** 


### NURSING PROGRESS AND ITS DEVELOPMENTS.

### VI. The Probationer's First Two Years.

IN all but one of the British training-schools which can claim to be considered in the first class the period of instruction given to the probationer before she rises to the status of a staff nurse is two years. In the London Hospital it is reduced to eighteen months. The widest possible variation exists in regard to the theoretical instruction given in lectures and classes no less than in the practical experience of nursing afforded to the probationers in the wards. Usually the lectures are divided

**NURSING PROGRESS**—(*continued*).

into two groups, for junior and senior students respectively. Sometimes examinations take place at the end of each year; but in many hospitals the probationers are not subjected to examination after the first period of nine months or a year, until they go up for their certificate at the end of the three years' course. The subjects taught show a certain conformity to a generally undefined standard of things which a trained nurse ought to know about the diseases from which patients ordinarily suffer. But there is no agreement among either doctors or matrons in regard to what constitutes an irreducible minimum of knowledge on such matters. Even when outside examiners are introduced, and this is gradually becoming the rule in the more advanced training-schools, the examiner seldom or never has any formal authority to revise the curriculum, but must examine on the syllabus presented to him.

One of the first duties imposed upon the College of Nursing by its establishment is the sorting out of the tangled skein of the nurse's theoretical education in order to bring into evidence what things can with most profit be assimilated by the student in the early stages of her probation, and in what manner the teaching thus imparted can best be reinforced and recapitulated before her training ends. For it is a common experience that sadly little of the lessons learnt during the struggling months of a first initiation into the mysteries of nursing is remembered to the end. There is an urgent call for an authoritative, but yet elastic, syllabus of theoretical instruction, drawn up with due regard to the fact that the probationer, student though she be, is yet also a part-time worker with her day all but filled by intensely engrossing and imperative duties. None but matrons living in the atmosphere of modern training-schools can ensure that the syllabus thus framed shall be really workable.

What practical opportunities should the training-school afford to each nurse in the practice of her nursing duties? Some period, varying according to the hospital, of from one to three months, in medical and surgical, male and female wards respectively, is the foundation on which nursing experience is built, and in county hospitals of the ordinary type every probationer goes through the mill much in the same rotation. But in larger, as well as in smaller, training-schools it is difficult, if not impossible, to ensure this equality of opportunity. A workable scheme of ward work which should be considered adequate for the training of the probationer would need to show perhaps a dozen variations before it could meet with acceptance as suited to the requirements of the work carried on in all the very different institutions which train probationers. The need for such a scheme has probably been felt by every newly appointed matron who finds herself for the first time arbiter of the destinies of nurses in respect to their measure of opportunity for learning their business. The subject bristles with difficulties. To what extent may training in special wards count as part of the general curriculum? They must, of course, be manned with junior probationers as well as the rest. How much time is it legitimate for a probationer to spend, say, in the children's or cancer wards? Again, what period of the probationer's first two years should be spent on night duty? How reconcile the claims of medical students and of probationers in the matter of dressings, and how afford the latter the practice they need in hospitals with a medical school? How supplement scanty opportunities for work in the theatre or elsewhere? There is no one answer to these and kindred queries, but there is a vast field of almost unexplored wisdom waiting to be garnered. Matron after matron has made her own experiments, registered her own triumphs, formed a workable system, trained a generation of good nurses, and passed away, leaving it to her successors to imitate (or perchance to undo) her life work. It is time that this rich harvest of experience was stored for the use of the whole nursing profession. There are wise heads already grappling with the difficulties which now beset matrons collectively and individually. And when the College of Nursing comes into its own it will be in a position to offer alternative solutions to the many problems which perpetually puzzle matrons in their task of training good nurses.

☛ Previous articles appeared December 8, 15, 22, and January 5 and 12.

